# Waste of Life

**Published:** 1875-04

**Authors:** P. H. Hayes


### THE WASTE OF LIFE.

P. H. HAYES, M. D.

People perish for lack of knowledge. A country practitioner said to me a week ago, "I have had sixty-seven cases of scarlet fever this winter, and I can trace them all to a man who came from the East and brought the contagion in his hair. Yet how many of those whose olive plants have withered and fallen under this infectious disease find their comfort in a kind but 'Mysterious Providence.'" But it is not always a mysterious Providence, unless it is always a mysterious providing of nature that cause and effect cannot be separated. If the same parties were to ask Tom Jones, in jail, "How came you here?" and he should reply, "Don't know, Mysterious Providence, I suppose," they would laugh in his face, and say, "you stole, Tom, you broke the law, Tom."

Mr. Smith's daughter lay sick, last summer for many weeks with a typhoid fever, and after a time a son was taken. He mourned his lot and thought how strangely Providence had dealt with him, until some one who believes that every event has some adequate cause, asked Mr. Smith how about the drains leading from his house and the purity of the water in the well. Yes, he did remember that just before Nettie was taken sick the drain got stopped up and it was a good while before he could get a man to open it again; and he remembered what a smell there was back of the house; and that for a time the well water did not taste good.

It is but a little more than a year ago that a doctor in Massachusetts put this question to forty-nine other physicians practicing in the agricultural districts of the commonwealth. What diseases are the most prevalent among the Farmers? Of forty-nine doctors,

| | |
|---|---|
| Rheumatism is mentioned by | 28 |
| Typhoid Fever by | 23 |
| Catarrh, Bronchitis and Consumption | 23 |
| Pneumonia by | 12 |
| Dyspepsia by | 10 |

Will the time ever come when men and women will be educated to know that not one of these diseases, nor any other, is without its own specific and appropriate cause, that every disease has its own run and is true to its own kind, and cannot, nevertheless, grow without it finds a soil in our blood? When will men find out that they may if they will be as expert in the care of their health as they now are in the management of their farms and their merchandise, and call their physician, not as an arbitrator but as a counsellor.

An inflamed eye cannot say, "surely the light is sweet." A fevered brain is only pained by the most beautiful musical sounds, a rheumatic man does not delight in walking, and what matters it if a man has cattle on a hundred hills, if the gout has its wrench upon him, or that he has houses and lands and friends innumerable, if he must have dyspepsia, be billious and gloomy. So, whatever our wealth in houses and lands, in wife, children and friends, in merchandise, gold, silver or jewels, there is forever, one underlying condition, without which, no man can have *health.*

A world without sickness is not a pure idealism, for in all the book of Genesis, covering a period of more than two thousand years, the word sick or sickness does not occur but once, and that at the very close of the book. And so unnatural and unheard of a thing was it that a son should die before his father that the only and single instance known is recorded. "And Haran died before his father Terah in the land of his nativity in Ur, of the Chaldees." The human constitution left the hand of its Maker with such superabounding life that the ages of men went up to hundreds of years. So thoroughly has all conception of such life as this died out from among men that we sometimes question whether it be possible that these old antediluvians could have measured time as we now do. Yet such is the native vigor of the human constitution, and such the inwrought divinity of body and soul that I firmly believe the race would rapidly recover all it has lost by a loving obedience to the laws of life.

How strange, that in our life of boasted intelligence there should be eighty thousand doctors, men, by education, set apart to meet a vast necesity, to take our lives in their hands when we are sick, and to whom sickness is business-wise, a bounty and a reward! Why not turn the tables and pay our physician to keep us well? What a revenue in health, life, enjoyment, in purse if you will, would these doctors bring us if their rewards were no longer the revenues of suffering, but the income from life and enjoyment.

Why should a doctor not have his parish and preach regularly, warning the people against the sins of the body, proclaiming the laws of health. His would be a law without a gospel, for there is no forgiveness, whoever sins here has no mediator, himself alone must expatiate his transgressions and he who obeys shall immediately and continually add life to joy and new joy to life. How few men and women we meet who are more than half alive, they are dyspetic or rheumatic, they have colds, coughs and congestions, they have poor blood and neuralgia, they are billious and gloomy. Has God created men to this estate? Never!

No disease is so common as dyspepsia, yet not another organ in the body is so "*tough*" as the stomach. It will endure alcoholic stimulants, opiates, drugs innumerable, it will undertake to digest rank and greasy preparations of food, dishes made up and compounded to the last degree, and yet after years of such abuse will often be a good stomach still and turn round and renew the life and stave off premature old age, if we will but take away these galling burdens.

Nine-tenths of all that disguised struggle between life and death which we call sickness, chronic disease, or infirmity is but dyspepsia, reporting itself in every organ by the poor blood it sends there and by its morbid sympathies through the nervous system. Thus the man who has dyspepsia in the stomach has dyspepsia in the brain, in the heart, in the lungs, in the joints, and in his temper and tone of mind. Is not the stomach the spring of the blood, can blood full of life, come from a dyspeptic stomach, must not every organ live upon this blood and give out a life in kind as it receives, even to the brain itself, the organ of all the faculties and aspirations of the soul? How many times have patients come to me with apparantly as many diseases as they have organs in their bodies, and this was no mystery to me, for was not every organ instantly feeding upon a bad blood, and sending forth an outcry of rebellion in the form of pain, ache or weakness, all translatable into one single expresssion "more and redder arterial blood." How often is our blood born to poverty in the making and then poltuted by breathing a bad air or by drinking impure water or by indiscriminate and nostrum dosing. Shakespeare, who knew everything describes blood-poisoning as though from polluted air or water.

> "Whose effect
> Holds such an emnity with the blood of man,
> That swift as quicksilver it courses through
> The natural gates and alleys of his body."

And the inimitable plea of Othello to Desdemona pressing his suit upon the quality of his blood.

> "Mislike me not for my complexion,
> The shadowed livery of the burnished sun,
> Bring me the fairest creature northward born,
> And let us make incision for your love,
> And prove whose *blood is reddest*,
> His or mine."

